# Targeted alpha therapy with astatine-211-labeled anti-PSCA A11 minibody shows antitumor efficacy in prostate cancer xenografts and bone microtumors

**DOI:** 10.1186/s13550-020-0600-z

**Published:** 2020-02-11

**Authors:** Tom A. Bäck, Karin Jennbacken, Malin Hagberg Thulin, Sture Lindegren, Holger Jensen, Tove Olafsen, Paul J. Yazaki, Stig Palm, Per Albertsson, Jan-Erik Damber, Anna M. Wu, Karin Welén

**Affiliations:** 1 Department of Radiation Physics, Institute of Clinical Sciences, University of Gothenburg, Gula stråket 2B SE-413 45, Gothenburg, Sweden; 2 Department of Urology, Institute of Clinical Sciences, Sahlgrenska Cancer Center, University of Gothenburg, Gothenburg, Sweden; 3 PET and Cyclotron Unit, KF-3982, Rigshospitalet, Copenhagen, Denmark; 4 Department of Molecular Imaging and Therapy, Beckman Research Institute of the City of Hope, Duarte, CA USA; 5 Department of Oncology, Institute of Clinical Sciences, University of Gothenburg, Gothenburg, Sweden; 6 Department of Oncology, Sahlgrenska University Hospital, Region Västra Götaland, Gothenburg, Sweden; 7 Bioscience Cardiovascular, Early Cardiovascular, Renal and Metabolism, BioPharmaceuticals R&D, AstraZeneca, Gothenburg, Sweden

**Keywords:** Metastatic prostate cancer, Targeted alpha therapy, Alpha particles, Alpha-radioimmunotherapy, Astatine-211, Prostate stem cell antigen, Intratibial microtumors

## Abstract

**Purpose:**

Targeted alpha therapy (TAT) is a promising treatment for micrometastatic and minimal residual cancer. We evaluated systemic α-radioimmunotherapy (α-RIT) of metastatic castration-resistant prostate cancer (mCRPC) using the α-particle emitter ^211^At-labeled to the anti-PSCA A11 minibody. A11 is specific for prostate stem cell antigen (PSCA), a cell surface glycoprotein which is overexpressed in more than 90% of both localized prostate cancer and bone metastases.

**Methods:**

PC3-PSCA cells were implanted subcutaneously (s.c.) and intratibially (i.t) in nude mice. Efficacy of α-RIT (two fractions—14-day interval) was studied on s.c. macrotumors (0, 1.5 and 1.9 MBq) and on i.t. microtumors (~100–200 μm; 0, 0.8 or 1.5 MBq) by tumor-volume measurements. The injected activities for therapies were estimated from separate biodistribution and myelotoxicity studies.

**Results:**

Tumor targeting of ^211^At-A11 was efficient and the effect on s.c. macrotumors was strong and dose-dependent. At 6 weeks, the mean tumor volumes for the treated groups, compared with controls, were reduced by approximately 85%. The separate myelotoxicity study following one single fraction showed reduced white blood cells (WBC) for all treated groups on day 6 after treatment. For the 0.8 and 1.5 MBq, the WBC reductions were transient and followed by recovery at day 13. For 2.4 MBq, a clear toxicity was observed and the mice were sacrificed on day 7. In the long-term follow-up of the 0.8 and 1.5 MBq-groups, blood counts on day 252 were normal and no signs of radiotoxicity observed. Efficacy on i.t. microtumors was evaluated in two experiments. In experiment 1, the tumor-free fraction (TFF) was 95% for both treated groups and significantly different (*p* < 0.05) from the controls at a TFF of 66%). In experiment 2, the difference in TFF was smaller, 32% for the treated group versus 20% for the controls. However, the difference in microtumor volume in experiment 2 was highly significant, 0.010 ± 0.003 mm^3^ versus 3.79 ± 1.24 mm^3^ (treated versus controls, respectively), i.e., a 99.7% reduction (*p* < 0.001). The different outcome in experiment 1 and 2 is most likely due to differences in microtumor sizes at therapy, or higher tumor-take in experiment 2 (where more cells were implanted).

**Conclusion:**

Evaluating fractionated α-RIT with ^211^At-labeled anti-PSCA A11 minibody, we found clear growth inhibition on both macrotumors and intratibial microtumors. For mice treated with multiple fractions, we also observed radiotoxicity manifested by progressive loss in body weight at 30 to 90 days after treatment. Our findings are conceptually promising for a systemic TAT of mCRPC and warrant further investigations of ^211^At-labeled PSCA-directed vectors. Such studies should include methods to improve the therapeutic window, e.g., by implementing a pretargeted regimen of α-RIT or by altering the size of the targeting vector.

## Introduction

The high energy, short-range α-particles are suitable in therapies of minimal residual and small-size disease, including widespread small-sized metastatic disease in the bone. Compared with treatments using beta-emitters, targeted alpha therapy (TAT) could minimize surrounding tissue toxicity and increase the radiation energy specifically delivered to tumor cells. The α-particles’ characteristics have been advantageously exploited for palliative treatment of metastatic castration-resistant prostate cancer (mCRPC) with ^223^Ra-dichloride [1] which targets the cancer cells indirectly through accumulation in the bone. Utilizing direct targeting in TAT, e.g., by alpha-radioimmunotherapy (α-RIT), could further leverage α-particle-based treatments of mCRPC through increased efficacy and broadened indication. Studies have shown promising results using targeting of the prostate-specific membrane antigen (PSMA) in combination with the α-emitters ^225^Ac [2–6], ^213^Bi [7] and ^211^At [8, 9]. While the results from reported and ongoing anti-PSMA-studies have been very encouraging, these reports have also pointed out limitations; some mCRPC patients have a low PSMA-expression [10], a fraction of pathologic positive lesion sites can be PSMA-negative [11], i.e., there is heterogeneity in the occurrence and expression of PSMA. This underlines the need for other targets complementary to PSMA. Prostate stem cell antigen (PSCA) is a cell-surface antigen expressed in normal prostate and overexpressed in prostate cancer (PC) tissues. It is detected in ~90% of primary prostate cancers and the expression increases with high Gleason score, advanced stage and bone metastasis [12], i.e., PSCA is a putative target for targeted therapy of mCRPC [13].

We studied efficacy of α-RIT on mCRPC in mice, mimicking microscopic bone metastases, using an 80 kDa anti-PSCA antibody fragment (A11 minibody) [14, 15], labeled with the α-emitter ^211^At. A minibody is expected to have a fast pharmacokinetics relative to larger or full-sized antibody formats and thus could be a therapeutically favorable match with the short-lived ^211^At (half-life 7.2 h). Efficient targeting of the ^211^At-A11 minibody on PSCA-expressing tumors was demonstrated. Following biodistribution and blood toxicity studies, we conducted fractionated systemic TAT and observed strong antitumor efficacy on both subcutaneous (s.c.) macrotumors and intratibial (i.t.) microtumors. This indicates that directly targeted TAT by α-RIT is a promising regimen for treatment of mCRPC and that further studies of ^211^At-labeled agents are warranted, including anti-PSCA vectors like the anti-PSCA A11 minibody.

## Methods

### Minibody A11

The humanized anti-PSCA A11 minibody (single-chain Fv-CH3 dimer, 80 kDa) was produced as described before [13, 14, 16]. Briefly, the A11 minibody (affinity 16 nM) was produced and affinity matured from the parental humanized anti-PSCA antibody fragment hu1G8 minibody, which in turn was derived from the 1G8 monoclonal antibody.

### PSCA-expressing PC3 cell-clone

The castration-resistant prostate cancer cell line PC3 (European Collection of Cell Cultures (Wiltshire, UK) was cultivated in RPMI-1640 (PAA Laboratories, Linz, Austria) supplemented with glucose, sodium pyruvate, 1% penicillin-streptomycin (Invitrogen, Carlsbad, CA), and 10% fetal bovine serum (FBS, Invitrogen).

To establish a PSCA-expressing sub-clone, PC3 cells were transfected with ScaI-linearized pcDNA3.1 comprising the full-length human PSCA gene [17]. Stably transfected clones were isolated, expanded and screened for PSCA-expression by RT-qPCR (Additional file 4: Figure S4a). High-expression clones were evaluated for changes in cell proliferation with CyQuant (Invitrogen). The final screening of PSCA-PC3 clones was made by a cell-binding assay using radiolabeled A11 minibody (Additional file 4: Figure S4b), as described below.

### Astatine-211, radiolabeling and immunoreactivity

Astatine-211 was produced at the PET and Cyclotron Unit, Copenhagen University Hospital, Denmark. Dry distillation and radiolabeling were performed as described before [18, 19]. Prior to ^211^At-labelling, the A11 minibody was modified via conjugation with m-MeATE (*N*-succinimidyl 3(trimethylstannyl) benzoate. The resulting immunoconjugate was then labeled with ^211^At. A dry astatine residue (50–100 MBq) was first activated by adding *N*-iodosuccinimide in methanol:1% acetic acid. Then the immunoconjugate (100 μg) was added and reacted with the astatine for 60 s. The radiolabeled antibody fraction was isolated by passage over a NAP-5 column (GE Healthcare) eluted with PBS. Radiochemical purity of ^211^At-A11 was analyzed by methanol precipitation. Stability and fragmentation analysis was performed at 4 h after radiolabeling, using size exclusion liquid chromatography, FPLC (Superdex 200, GE Healthcare, Sweden). Immunoreactive fraction (IRF) was determined using a cell assay. Serial 1:2-dilutions of PC3-PSCA cell suspensions (0.15625–10 million cells/mL) were incubated with ^211^At-A11 (5 ng) at 8 °C for 3 h. After centrifugation and cell pellet washing, the IRF was calculated from the double-inverse plot of specific binding (B/T) versus cell concentration. Injection solutions were prepared by diluting ^211^At-A11 in PBS.

### Biodistribution and uptake in macrotumors

Male nude mice (BALB/c nu/nu, 8 weeks old; Charles River) were inoculated s.c. with PC3-PSCA tumor cells (2 million in 200 μL medium) on the flank. The mean tumor volume at the study start was ~200 mm^3^. At tumor volumes > 1300 mm^3^ or body weight loss > 20%, mice were taken out of the study and sacrificed. This study was approved by the Gothenburg Ethical Committee for Animal Research (Ethical permit: 283-2011), and all animals were maintained as regulated by the Swedish Animal Welfare Agency. For biodistribution, 20 mice were injected intravenously (i.v.) in the tail with ^211^At-A11 (260 ± 20 kBq in 0.12 mL PBS). At 1, 5, 9, 23 and 42 h post-injection (hpi), 4 mice per time point were sacrificed and the organs dissected, weighed and measured for ^211^At activity using a gamma counter (Wizard 1480, Perkin Elmer). An extra cohort of 6 mice was used to investigate the effect (at 5 and 9 hpi) of pre-treatment with sodium perchlorate (NaClO_4_) to reduce uptake of free ^211^At in certain organs. The NaClO_4_ (Sigma Aldrich Sweden AB) was injected intraperitoneally (1.2 μmol/g in 0.1 mL PBS) 24 h and 1 h before injection of the ^211^At-A11 minibody.

At 1 and 5 hpi, bone marrow samples from the femoral bones were taken to measure the uptake in the bone marrow (BM). The uptake of ^211^At (percentage of injected activity per gram tissue, %IA/g) was corrected for radioactive decay to the injection time. The BM-to-blood-ratio (BMBLR) was calculated by dividing %IA/g for BM by that of the blood. Since a secure dissection of the thyroid is prone to error, we instead dissect the corresponding part of the throat. Anatomically, this part contains the thyroid, larynx, a part of the trachea and the related connective tissues. For estimation of uptake and absorbed dose to the thyroid, we then assumed that all activity measured for the throat was contained in the thyroid (using a standard weight of 3 mg).

### Dosimetry

The cumulated activity (total number of decays, *Ã*) for each organ was calculated from time-activity plots (activity per gram versus hpi) of the biodistribution data without NaClO_4_ pre-treatment. Including contributions only from α-particles, the mean absorbed dose (D) was calculated as:

$$ D=\frac{\widetilde{A}}{m}{\varDelta}_{\alpha }{\varphi}_{\alpha } $$

where *m* denotes tissue mass, *φ*_*α*_ the absorbed fraction (set to 1) and Δ_α_ the mean energy per ^211^At decay (1.09 × 10^−12^ J). Finally, the absorbed dose for each tissue was calculated as the sum of all injections. For biodistribution with NaClO_4_ pre-treatment, biodistribution data is presented for 5 hpi and 9 hpi. To approximate absorbed doses following pre-treatment with NaClO_4_ , theoretical biodistribution data were calculated for time points 1, 23 and 42 hpi. This was done by using the quotient of the %IA/g-values received with pre-treatment divided by the %IA/g-value without pre-treatment. The quotient found at 5 hpi, for each organ respectively, was used to calculate a theoretical %IA/g-value for the 1 hpi time point, and the quotient for 9 hpi was used for the 23 hpi and 42 hpi time points.

### Myelotoxicity after alpha-RIT

Considering the bone marrow as the primary dose-limiting organ, a separate cohort of mice was used to estimate the maximum tolerable activity (MTA) in the therapy studies. The mean absorbed dose (Gy/MBq) to the BM was calculated from the %IA/g in the blood using the BMBLR at 5 and 9 hpi. Three groups (5 mice per group) were i.v.-injected with 0.8, 1.5 or 2.4 MBq of ^211^At-A11 (corresponding to BM absorbed doses of 1, 2 and 3 Gy, respectively). The cell count for white blood cells (WBC), red blood cells (RBC) and platelets (PLT), as well as the hemoglobin (HGB), was measured before (day 1) and after treatment (day 6 and 13), by tail vein blood samples analyzed using a microcell counter (F-820; Sysmex, Kobe, Japan).

### Alpha-RIT of macrotumors

For α-RIT of s.c. macrotumors, the animals were pre-treated with NaClO_4_ (twice before each treatment, described above) and then treated twice, 14 days apart, allowing for BM recovery in-between treatments. Three weeks after cell implantation (tumor volume ~200 mm^3^), the ^211^At-A11 was i.v.-injected (1.5 or 1.9 MBq in 0.15 mL PBS, 10 mice per group). A control group (20 mice) received non-radiolabeled A11 minibody. Tumor growth and body weight was monitored weekly up to 120 days after therapy. The tumor volume (*V*) was determined from the largest (a) and the perpendicular diameter (b) and calculated as: *V* = (a × b^2^)/2.

### Alpha-RIT of microtumors

Efficacy of α-RIT with ^211^At-A11 was studied in two separate independent experiments. Bone microtumors were established by intratibial (i.t.) injections (right leg tibiae) with PC3-PSCA cells under anesthesia, as previously described [20]. After implantation, the animals received analgesics (Rimadyl; 5 mg/kg) for 5 days. Alpha-RIT of microtumors was conducted in two experiments (pre-treatment with NaClO_4_ as described above). In experiment 1, 63 mice were given 20,000 PC3-PSCA cells. Seven days later, the mice were divided into 3 groups and the treatment started. Group 1 (*n* = 19) and 2 (*n* = 20) were i.v.-injected with ^211^At-A11 (0.8 MBq and 1.5 MBq, respectively, in 0.15 mL PBS). A control group (*n* = 24) was given non-radiolabeled A11 minibody. The same treatments were repeated 14 days later. Six weeks after the first treatment, the mice were sacrificed, the tibiae dissected, fixed in paraformaldehyde (3 days), decalcified in Parengy, serially sectioned (4-μm thickness), and stained with hematoxylin and eosin (Histocenter AB, Gothenburg, Sweden). Tumor growth was evaluated from the H&E slides in terms of presence or absence of tumor cells.

In experiment 2, the injected number of cells was 5 times higher than in experiment 1, a total of 100,000 cells was given. At therapy, 7 days later, a cohort of 5 untreated mice was sacrificed to estimate the microtumor sizes at treatment. The treated group (*n* = 19) received two i.v. injections of ^211^At-A11 (1.5 MBq in 0.15 mL PBS) 14 days apart. The control group (*n* = 20) received unlabeled A11 minibody. Six weeks after treatment, the tumor growth was determined by histological analysis, as described above. The tibiae were serially sectioned throughout, corresponding to ~200 sections per tibia, depending on the sectioning angle. Each microtumor volume was estimated from the serial sections using multiple sections (4 μm to 100 μm apart, depending on tumor size). The tumors were approximated as an ellipse and the major, a, and minor, b, (perpendicular) semi-axes were measured. For each section, the tumor area, A, was calculated as A = a × b × π, and then the volume by multiplication with section thickness. Finally, the tumor volume was calculated by summing all sections encompassing the whole microtumor.

### Statistical methods

Difference in organ uptake after biodistribution with and without NaClO4 pre-treatment was studied at two time points, 5 and 9 hpi, and analyzed (each time point respectively) using the unpaired Student’s *t* test. For α-RIT on i.t. microtumors, the differences in tumor volume after treatment between independent groups were evaluated with Mann–Whitney *U* test. Differences in tumor frequencies between treated and control groups were analyzed with the Pearson’s chi-squared test. *P* values < 0.05 were considered as statistically significant.

## Results

### Radiolabeling and immunoreactivity

The radiochemical purity of ^211^At-A11 minibody was > 95%. Liquid chromatography after radiolabeling showed no aggregation or fragmentation, and less than 0.4% free astatine at 4 h after labeling (Additional file 1: Fig. S1). The IRF was 0.6–0.7 (Additional file 2: Fig. S2), i.e., in agreement with previous data [14].

### Biodistribution and uptake in macrotumors

**Fig. 1:**
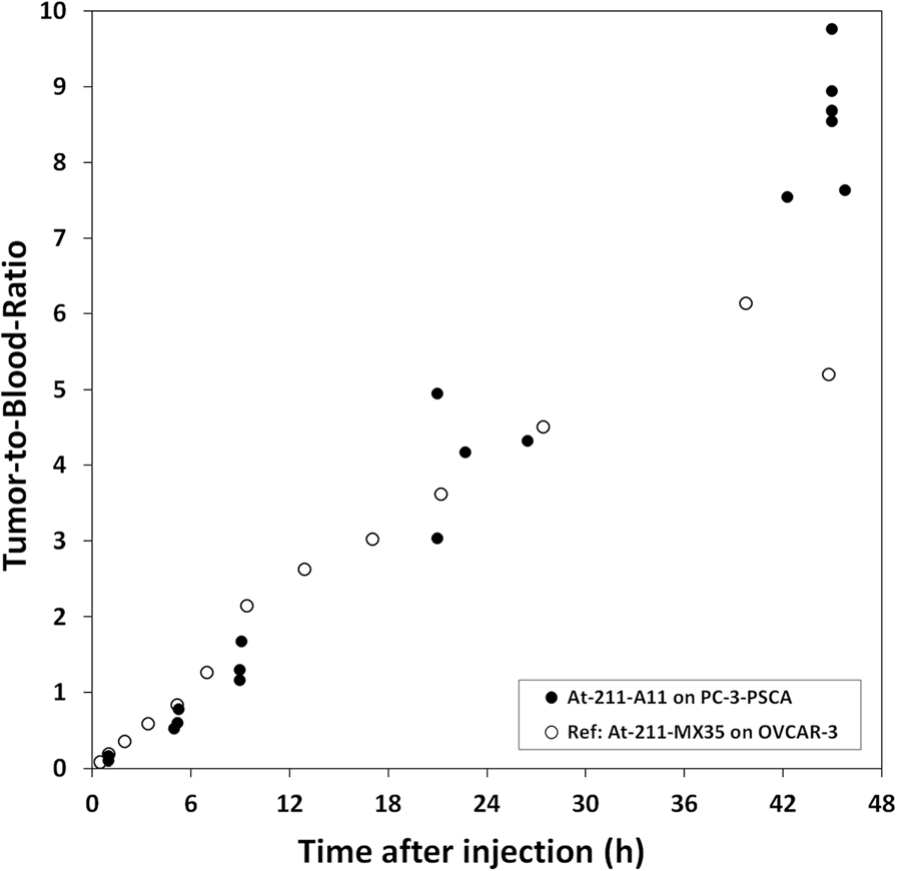
The tumor-to-blood ratio (of %IA/g) of ^211^At-A11 on s.c. PC3-PSCA macrotumors plotted versus time after injection. Included for comparison is the tumor-to-blood ratio of ^211^At-MX35-F(ab’)_2_, which was shown to be therapeutically efficient on macrotumors in previous studies of ovarian cancer [21]

**Fig. 2:**
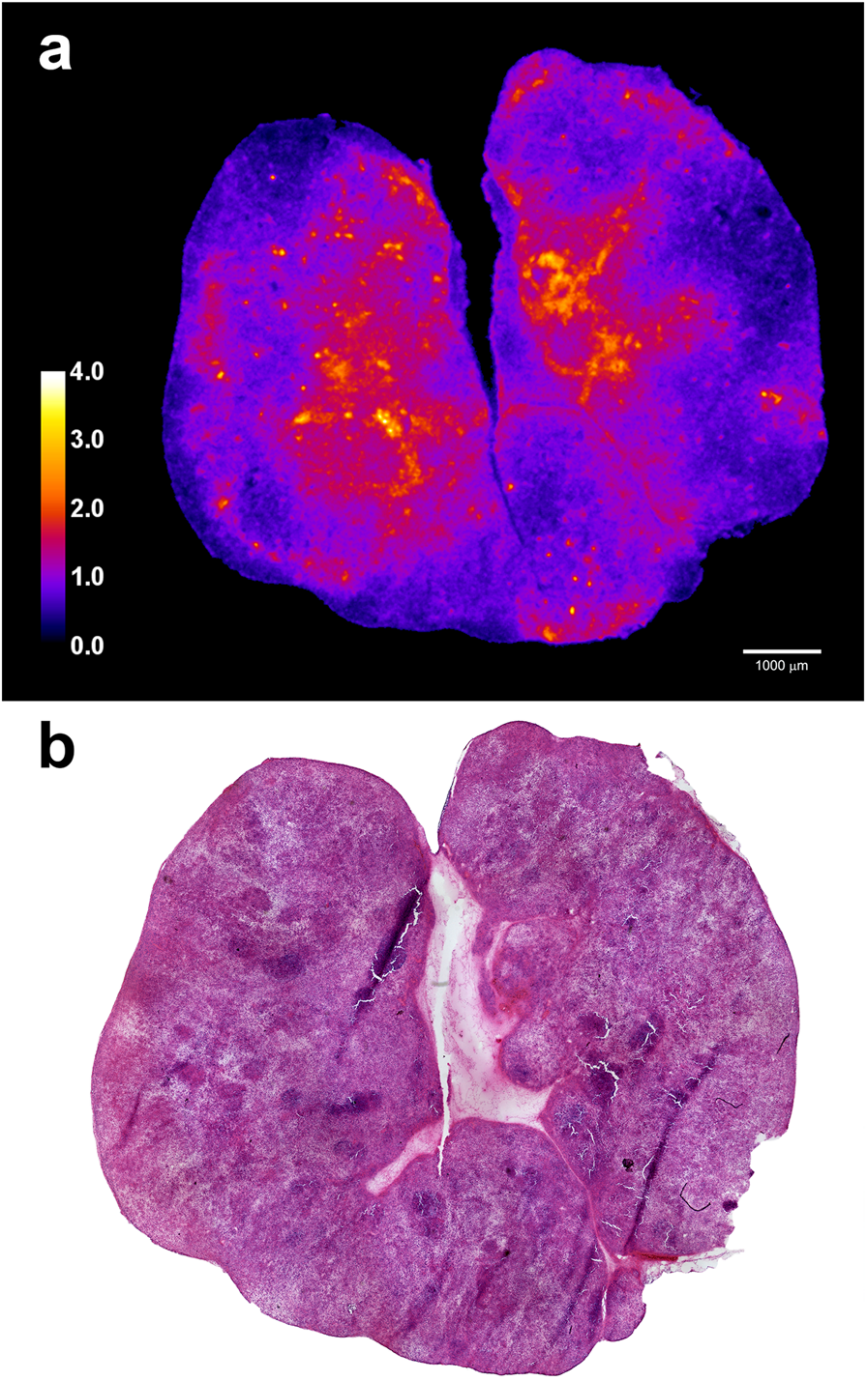
The intratumoral activity distribution of ^211^At-A11 in s.c. PSCA-PC3 macrotumors at 3 hpi (**a**) as studied by alpha camera imaging [22]. The color coded LUT is normalized so that 1.0 represent the mean activity for the whole tumor section. White scale bar indicate 1000 μm. Consecutive H&E-stained section (**b**). *LUT* look-up-table; *H&E* Hematoxylin and eosin

**Fig. 3:**
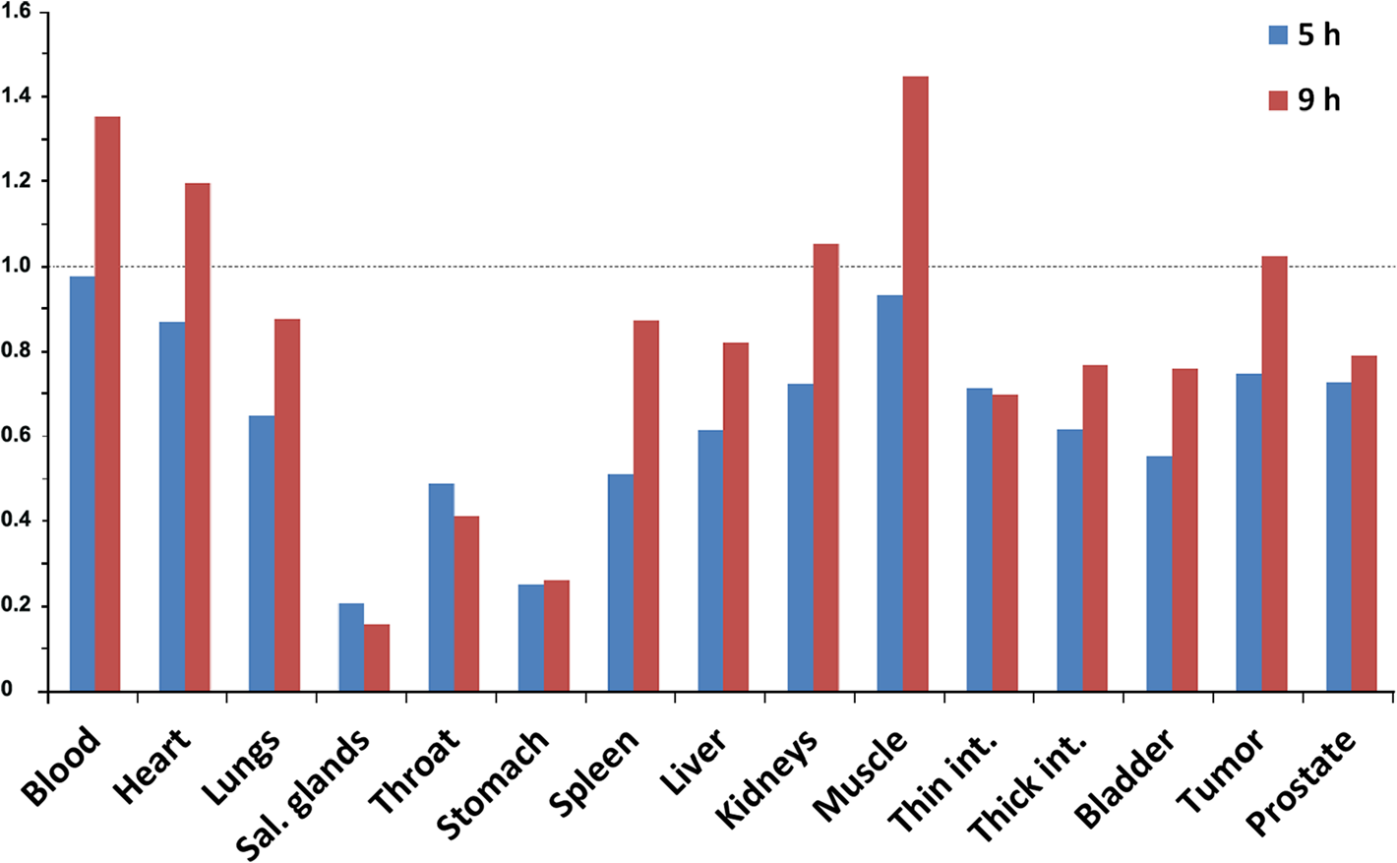
The relative effect of pre-treatment with sodium perchlorate (2.5 mg NaClO_4_·H_2_0) on the biodistribution of ^211^At-A11 minibody. For each organ. the %IA/g-value received with pre-treatment was divided with the %IA/g-value without pre-treatment, respectively for the two time points

Biodistribution data of ^211^At-A11 is presented in Table 1. The uptake in PC3-PSCA macrotumors was fast, maximizing at 7.2%IA/g after 5 h, and then decreasing slowly to 5.6 %ID/g at 23 hpi. Blood clearance was relatively fast, as expected for a minibody. The tumor-to-blood ratio increased with time (Fig. 1), reaching a ratio of 5 at 23 hpi and close to 10 at 42 hpi. Included in the plot for comparison is the tumor-to-blood ratio of ^211^At-MX35-F(ab’)_2_, for which complete eradication of macrotumors was found by fractionated systemic α-RIT in an ovarian cancer model [21]. Alpha camera imaging [22] of the intratumoral activity distribution at 3 hpi showed that ^211^At-A11 was well distributed throughout the macrotumors (Fig. 2), with hot-spots corresponding to the vasculature. Pre-treatment with NaClO_4_ decreased the uptake in most organs (Fig. 3), and was therefore given in the therapies. For stomach, salivary glands, throat, lungs and spleen, the decrease (mean of 5 hpi and 9 hpi) was 74%, 82%, 55%, 24% and 31%, respectively. A statistical comparison between the groups with and without NaClO_4_- pre-treatment is included in Table 1. It can be noted that for salivary glands, throat and stomach, NaClO4-pretreatment gave a significant decrease in uptake at both 5 hpi and 9 hpi. For the liver, spleen and intestines, there was a significant decrease at 5 hpi, but not at 9 hpi. For the blood, there was no difference at 5 hpi, but at 9 hpi, there was a significant increase. For tumors, finally, no significant differences were observed, neither at 5 hpi nor 9 hpi.

**Table 1:** Biodistribution with ^211^At-A11 minibody

Organ	1 h	5 h	5 h (NaClO_4_)	*p* value	9 h	9 h (NaClO_4_)	*p* value	23 h	42 h	
Blood	20.1 ± 1.8	9.4 ± 0.9	9.1 ± 0.4	ns	3.2 ± 0.2	4.4 ± 0.6**	0.0090	1.3 **±** 0.1	0.3 **±** 0.0	
Bone marrow	5.6 ± 1.0	N.D.	2.7 ± 0.6	–	N.D.	N.D.	–	N.D.	N.D.	
Heart	6.7 ± 0.9	5.1 ± 0.7	4.4 ± 0.3	ns	2.3 ± 0.2	2.8 ± 0.5	ns	1.3 **±** 0.2	0.3 **±** 0.1	
Lungs	11.3 ± 0.3	11.3 ± 2.0	7.3 ± 1.1*	0.0284	6.4 ± 1.0	5.6 ± 0.9	ns	4.0 **±** 0.7	1.2 **±** 0.2	
Salivary glands	5.9 ± 0.7	14.7 ± 4.4	3.0 ± 0.7**	0.0067	13.4 ± 1.9	2.1 ± 0.3****	<0.0001	15.1 **±** 3.4	4.2 **±** 1.4	
Throat	10.3 ± 4.6	12.3 ± 3.1	6.0 ± 0.1*	0.0187	10.4 ± 2.0	4.3 ± 0.7**	0.0011	14.3 **±** 1.8	8.4 **±** 4.8	
Stomach	7.4 ± 2.1	17.4 ± 6.9	4.4 ± 0.7*	0.0251	15.7 ± 2.6	4.1 ± 1.1***	0.0002	13.7 **±** 2.5	2.1 **±** 0.9	
Liver	9.9 ± 0.6	5.1 ± 0.7	3.1 ± 0.4**	0.0087	2.3 ± 0.3	1.9 ± 0.3	ns	1.6 **±** 0.1	1.0 **±** 0.1	
Spleen	10.5 ± 1.5	9.7 ± 2.1	5.0 ± 1.2*	0.0181	4.8 ± 0.6	4.4 ± 1.2	ns	2.7 **±** 0.2	1.6 **±** 0.3	
Kidneys	9.6 ± 0.8	6.6 ± 1.1	4.8 ± 0.8	ns	2.8 ± 0.3	2.9 ± 0.6	ns	1.9 **±** 0.3	0.5 **±** 0.0	
Muscle	0.9 ± 0.2	0.9 ± 0.2	0.8 ± 0.2	ns	0.5 ± 0.1	0.8 ± 0.5	ns	0.3 **±** 0.0	0.1 **±** 0.1	
Small intestine	4.1 ± 1.3	3.7 ± 0.3	2.7 ± 0.2**	0.0024	2.4 ± 0.7	1.7 ± 0.3	ns	1.7 **±** 0.6	0.5 **±** 0.2	
Large intestine	2.0 ± 0.1	3.4 ± 0.5	2.1 ± 0.5*	0.0153	1.9 ± 0.1	1.5 ± 0.4	ns	1.3 **±** 0.3	0.3 **±** 0.2	
Bladder	4.3 ± 1.3	11.4 ± 6.3	6.3 ± 1.7	ns	7.5 ± 4.6	5.7 ± 3.0	ns	2.8 **±** 1.7	0.8 **±** 0.3	
Tumor	3.0 ± 0.2	7.2 ± 1.7	5.4 ± 1.5	ns	5.4 ± 2.5	5.6 ± 1.6	ns	5.6 **±** 1.0	2.1 **±** 0.6	
Prostate	3.6 ± 0.6	11.1 ± 0.9	5.0 ± 0.9	ns	4.5 ± 0.4	3.5 ± 1.0	ns	5.0 **±** 2.0	2.1 **±** 0.7	

Uptake data (%IA/g ± SD) at different times after i.v. injection of ^211^At-A11 minibody. At 5 and 9 hpi, data is presented with and without NaClO_4_ pre-treatment.(*n* = 4) and difference between the groups, at 5 and 9 hpi respectively, analyzed using unpaired Student’s *t* test: *p < 0.05, **p < 0.01, ***p < 0.001, ****p < 0.0001

%IA/g Percent of injected activity per g; *i.v.* Intraveneous; *ns* Not significantly different; *N.D.* Not determined

### Myelotoxicity and uptake in bone marrow

The BM uptake at 1 and 5 hpi corresponded to a BMBLR of 0.30 ± 0.03 and 0.28 ± 0.02 (mean ± SEM), respectively. Using a mean BMBLR of 0.29, the calculated mean absorbed dose to the BM was 1.3 Gy/MBq. Alpha-RIT resulted in clear reductions in WBC at day 6 for all treated groups (2.3 ± 0.4, 1.2 ± 0.3, and 0.7 ± 0.4 × 10^9^/L, mean ± SEM, for 0.8, 1.5 and 2.4 MBq, respectively) as compared with untreated controls (4.8 ± 2.1). At day 13, the WBC for the 0.8 and 1.5 MBq group (6.6 ± 0.5 and 6.2 ± 0.8, respectively) had recovered to a level similar to the controls (7.1 ± 1.3). The effect on PLT was similar to that of the WBC, with clear reductions at day 6 for the 2.4 and 1.5 MBq groups (Additional file 3: Figure S3). No effect was seen on RBC or HGB. For the 2.4 MBq group, several mice showed abnormal signs (dehydration and malnutrition) at day 7 and were sacrificed. All mice in the 0.8 MBq and 1.5 MBq groups were kept for long-term study of myelotoxicity. At day 252, no sign of radiotoxicity was seen and both groups had normal WBC counts (10.7 ± 3.1 and 9.8 ± 1.2, respectively). The values for PLT, RBC and HGB were also normal. The MTA of ^211^At-A11 minibody was estimated to be within 1.5–2.4 MBq.

### Dosimetry

Absorbed doses to organs and macrotumors were calculated from biodistribution data with and without NaClO_4_ pre-treatment and are shown in Table 2. Without NaClO_4_ pre-treatment, the highest doses were found for the thyroid, stomach, salivary gland, followed by the lungs, spleen and the bladder. The dose to blood was 4.7 Gy/MBq, corresponding to a BM dose of 1.3 Gy/MBq. The dose to macrotumors was 2.2 Gy/MBq. When pre-treatment with NaClO_4_ was given, the estimated absorbed doses decreased for most organs except the blood and bone marrow, for which a very small increase was observed, by 2% and 8%, respectively. For the salivary glands and stomach, the dose decreased by 82% and 75%, respectively and for the thyroid and spleen by 57% and 53%. A decrease in dose was found also for the bladder and lungs, by 36% and 27%, respectively. The dose to macrotumors decreased to 1.9 Gy/MBq, i.e., by 14% as did the prostate dose (37%).

**Table 2:** Mean absorbed organ doses from α-RIT with ^211^At-labeled A11 minibody

	Mean absorbed dose (Gy/MBq)	
Organ	Without NaClO_4_	With NaClO_4_^c^
Blood	4.7	4.8
Bone Marrow^a^	1.3	1.4
Heart	1.5	1.5
Lungs	3.3	2.4
Salivary glands	5.0	0.9
Throat	4.7	2.1
Thyroid^b^	64.7	27.5
Stomach	5.6	1.4
Liver	1.9	1.6
Spleen	2.8	1.3
Kidneys	2.1	1.7
Muscle	0.3	0.3
Small intestine	1.2	0.8
Large intestine	0.9	0.6
Bladder	2.8	1.8
Tumor	2.2	1.9
Prostate	2.4	1.5

^a^Dose to bone marrow was estimated using a bone marrow-to-blood-ratio of 0.29

^b^Dose to thyroid was estimated from the throat (assuming that all activity was located in thyroid and using a standard weight of 3 mg)

^c^Dose corresponding to pre-treatment with NaClO_4_ , using biodistribution data measured at 5 hpi and 9 hpi, complemented with theoretically calculated data for 1, 23 and 42 hpi

### Alpha-RIT on macrotumors

**Fig. 4:**
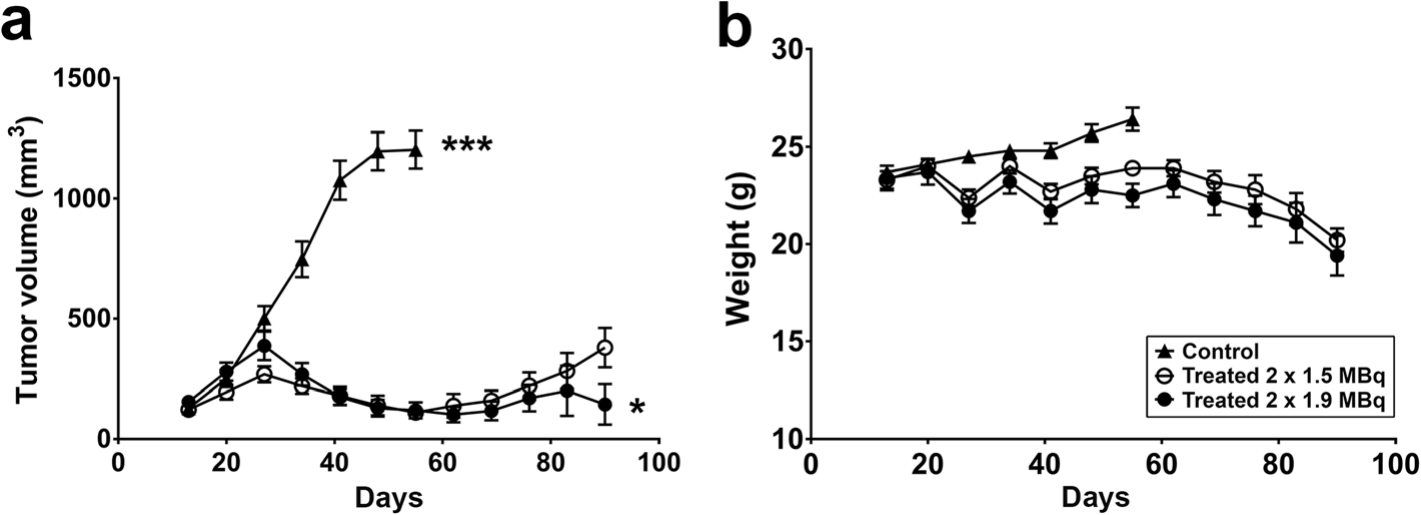
Treatment of s.c. PC3-PSCA macrotumors with different activities of ^211^At-A11. Tumor volumes (**a**) and mouse weights (**b**) are shown as mean ± SEM. ***p < 0.001, *p < 0.05

At treatment start, 20 days after implantation, the mean tumor volume was 240 mm^3^. Five weeks later (at day 55 after implantation), when 85% of the control mice were sacrificed due to exceeding tumor volume, the tumor volumes in both 1.5 MBq and 1.9 MBq groups (139 ± 41 mm^3^ and 128 ± 34 mm^3^, mean ± SD, respectively) were significantly different from the controls (*p* < 0.001; Fig. 4a). At the study end, 90 days after implantation, the difference between the 1.5 MBq and 1.9 MBq groups was only moderate but significant (*p* < 0.05), 379 ± 82 mm^3^ versus 143 ± 84 mm^3^, respectively. For all three groups, the age of the mice at the days mentioned above (20, 55 and 90) was 14, 19 and 24 weeks, respectively. At 70 days after treatment, both treated groups showed a clear weight loss (Fig. 4b).

### Alpha-RIT on microtumors

**Fig. 5:**
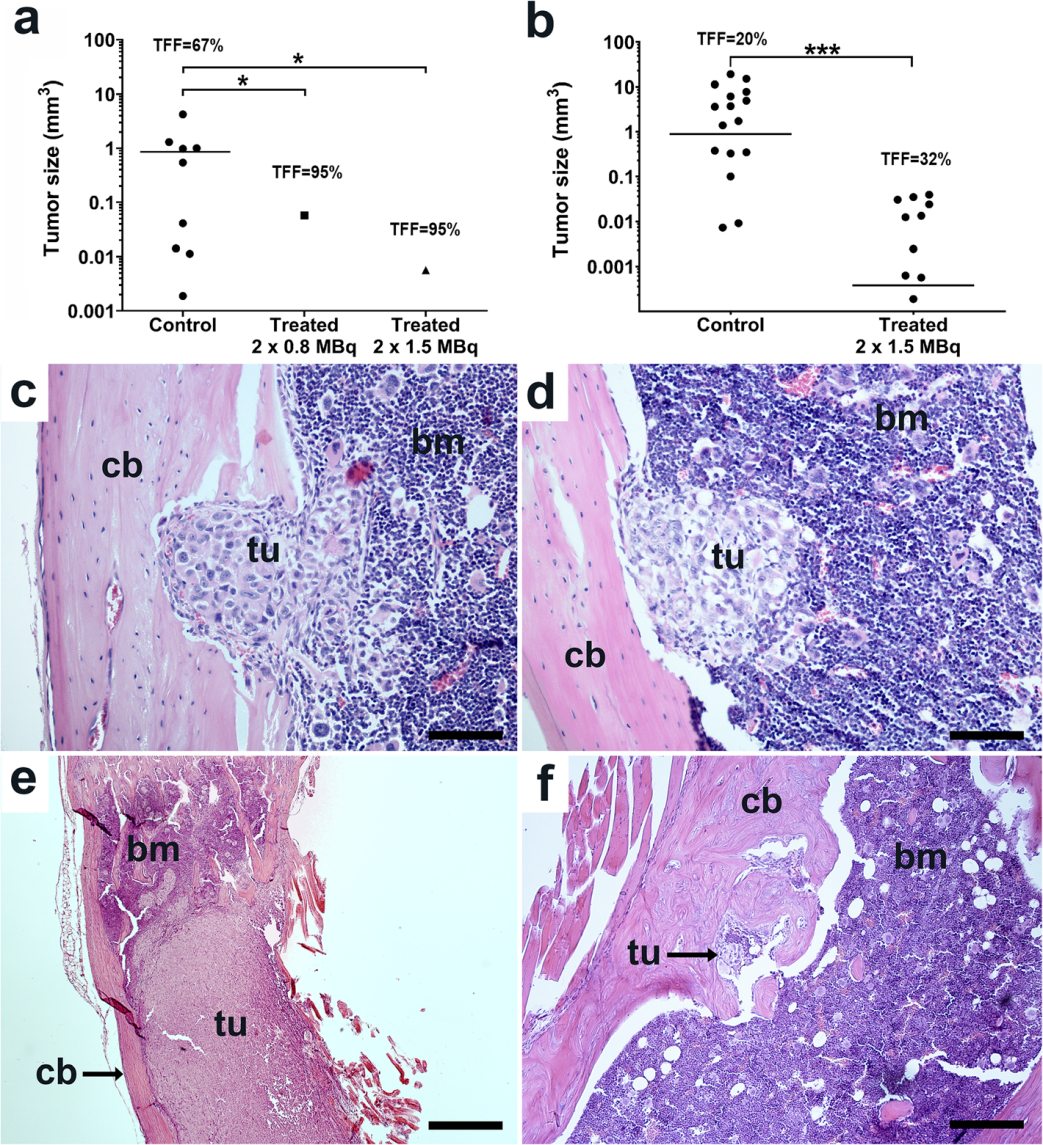
Effect of alpha-RIT with ^211^At-A11 on intratibial PC3-PSCA microtumors. Treatment was given twice two weeks apart. (**a**) Individual tumor sizes after treatment with ^211^At-A11 in study 1. Lines represent the mean size of identified tumors. *p < 0.05 Mann-Whitney *U* test. (**b**) Individual tumor sizes after treatment with ^211^At-A11 in study 2. ***p < 0.001 Mann-Whitney *U* test. Indicated is also the TFF for each group. (**c** and **d**) H&E staining of PC3-PSCA intratibial microtumors growing in the bone marrow and into the cortical bone at treatment start (7 days after cell implantation). Untreated tumor at end of experiment (**e**), and treated tumor (2 × 0.8 MBq) at end of experiment (**f**). Cortical bone (cb), bone marrow (bm), tumor (tu). Magnification 200×, black scale bar: 100 μm (**c** and **d**), magnification 40×, scale bar: 500 μm (**e** and **f**) *TFF* tumor free fraction

In experiment 1, the treated groups received two i.v. fractions with ^211^At-A11 (0.8 or 1.5 MBq mice,) starting at 7 days after cell implantation. Six weeks later, the number of mice with tumor-negative tibiae was 19 out of 20 and 18 out of 19, respectively, i.e., the tumor-free fraction (TFF) was 95% for both treated groups. For the controls (non-radiolabeled A11), the TFF was 66% (16 tumor-negative out of 24), i.e., also high, but statistically different from the treated (*p* < 0.05). The tumor volumes for the mice with tumors are shown in Fig. 5a. For the 8 control mice with tumors, the variation in tumor volume was large, with a mean volume of 0.89 ± 1.43 mm^3^. Only one single tumor per group was found for the treated mice, with volumes of 0.057 mm^3^ and 0.0056 mm^3^ (0.8 and 1.5 MBq, respectively).

In experiment 2, starting at 7 days after implantation, two fractions with ^211^At-A11 (1.5 MBq) were given and the results are shown in Fig. 5b. Five mice were sacrificed at treatment start and four of these (80%) had tumor-positive tibiae (Fig. 5c and d), with a mean tumor volume of 0.018 ± 0.12 mm^3^, corresponding ~100–400 μm diameter. At the study end, the TFF was 20% for the control group (non-radiolabeled A11) and 32% for the treated group, i.e., only a minor difference to the controls. However, the treatment had a strong effect on microtumor volume. The mean volume in the treated group was 0.015 ± 0.004 mm^3^, while for the untreated controls it was 4.74 ± 1.45 mm^3^, i.e., significantly larger (*p* < 0.001, Mann–Whitney *U* test) (Fig. 5c). This corresponds to a 99.7% decrease in microtumor volume. Examples of untreated and treated tumors at study end are shown in Fig. 5e and f, respectively.

## Discussion

Prostate cancer (PC) is the most common cancer among men in the Western world and the second in cancer-related death causes [23]. In the castration-resistant stage, current therapies prolong life with ~2–6 months [24] and new treatments are needed. Metastases remain the major clinical problem in the treatment of mCRPC, being the main cause of pain and death. Due to the α-particle characteristics, TAT is well suited for the treatment of metastatic disease. In contrast to ^223^Ra-dichloride, a regimen with direct targeting of PC cells, e.g., by PSCA-targeted α-RIT, could be effective on all types of PC micrometastases, i.e., both sclerotic and lytic bone lesions, distant metastases or remaining active cells in the prostate, as well on CTCs in the blood.

We investigated the efficacy of fractionated α-RIT with the anti-PSCA minibody A11, labeled with the α-emitter ^211^At. We found strong growth inhibition on both s.c. macrotumors and on intratibial microtumors. In experiment 1 on microtumors, the fraction of tumor-free mice (TFF) 95% for both treated groups, but also the untreated group had a relatively high TFF of 66%. Due to the relatively moderate difference between treated and untreated mice in terms of TFF, any treatment effect of ^211^At-A11 could be questioned. However, when we analyzed the efficacy in study 1 in terms of actual measured tumor volumes, we could observe a clear difference between the untreated controls versus both the treated groups (Fig. 5a). This difference was also statistically significant, both 0.8 MBq vs untreated and 1.5 MBq vs untreated. In experiment 2, the observed TFFs were markedly lower than in experiment 1, and the difference in terms of TFF was relatively small. But the difference in terms of mean tumor volume the difference was large and strongly significant, corresponding to a 99.7% decrease in microtumor volume for mice treated with ^211^At-A11. The difference in the outcome of the two experiments is most likely due to differences in microtumor sizes at therapy, or higher tumor-take in experiment 2 (where more cells were implanted). In experiment 1, the tumor size at therapy was unknown, but in experiment 2 the microtumor diameters ranged from 100 μm to 400 μm. The distribution and diffusion of ^211^At-A11 into the PC3-PSCA intratibial microtumors was not known. For microtumors with diameters > 150 μm (α-particle range ~70 μm), the ^211^At-A11 would need to be well distributed intratumorally if all cells should be irradiated and high TFF achieved. For pre-vascular microtumors, this is likely the case, but at the later stage, the intratumoral distribution can be negatively affected by heterogeneous vascularization [25].

For the s.c. macrotumors, the two treatments with ^211^At-A11 induced a clear reduction in tumor volume that was significantly different from untreated mice. However, the strong inhibition of tumor growth was also accompanied by radiotoxicity manifested as a clear and progressive reduction in body weight. This indicates the administered activities for therapy of macrotumors were very close to being above maximum tolerable. Indeed, the group receiving 2.4 MBq in the pre-study on myelotoxicity showed acute radiotoxicity and had to be sacrificed at day 7 after injection. The biodistribution study showed a fast uptake in macrotumors of the ^211^At-A11 minibody, maximizing at 5 hpi, and then decreased between 23 and 42 hpi. However, because of the 7.2 h half-life of ^211^At, 90% of the absorbed tumor dose was achieved already at 23 hpi. Since the clearance from blood was fast, this indicates that the fast kinetics of the minibody format could be a better match with the short-lived ^211^At, than would a full-sized IgG. Such comparison however, should also include smaller compounds, e.g., dia-, nano- or affibodies, i.e., could be addressed in future studies.

The observed growth inhibition of s.c. macrotumors indicated a favorable intratumoral activity distribution. Accordingly, alpha camera imaging [22] at 3 hpi revealed a more uniform distribution of the ^211^At-A11 minibody than was previously observed in an ovarian cancer xenograft model for the similarly sized ^211^At-MX35-F(ab)_2_ [22]. We have previously shown that complete eradication of s.c. macrotumors could be achieved by fractionated α-RIT for mean absorbed tumor doses > 10 Gy [21]. By adding a third fraction to the current study, i.e., increasing the highest tumor dose to 10.8 Gy, complete eradication might have been reached.

For the microtumors, the absorbed doses were unknown. However, estimations can be made from three different assumptions; (i) since the microtumors were residing in the BM cavity the doses would be at least the same as for the BM (1.3 Gy/MBq); (ii) weighing in antibody-to-tumor binding, the microtumor doses would be at least equal to that of the macrotumors (2.2 Gy/MBq), and (iii) since previous studies have shown that for the one and same cell line, the uptake in microtumors could be 20× higher than in macrotumors [26], i.e., a 20× higher absorbed dose for microtumors. Hence, for two injections of 1.5 MBq, these three scenarios would correspond to mean absorbed doses to the microtumors of 3.8 Gy, 6.6 Gy and 130 Gy, respectively. Tumor doses > 100 Gy have been observed for microtumors with a diameter of ≤ 100 μm [27].

For the normal organs, without NaClO_4_ pre-treatment given, the highest absorbed doses were found for organs known to accumulate free ^211^At, i.e., the thyroid, stomach, salivary gland and spleen. This could indicate catabolism of the labeled minibody or reduced in vivo stability of the ^211^At-labeling. Internalization of the minibody could contribute to the occurrence of free ^211^At, but PSCA is a slowly internalizing antigen [15]. As for the influence of radiolabeling, results from comparative biodistribution (Additional file 5: Fig. S5) showed that blood concentration was very similar for ^211^At-labeled and ^125^I-labeled (iodogen) A11 minibody, while the tumor uptake was lower for the ^125^I-labeled version. Since ^211^At-labeling of antibody fragments has been associated with reduced in vivo-stability we also compared the m-MeATE method to labeling with the boron cage B10 reagent (kindly provided by Dr. S. Wilbur, Seattle, US) [28], but for the latter, we observed an elevated uptake in the bone marrow at 1 hpi (Additional file 6: Fig. S6).

Pre-treatment with NaClO_4_ markedly reduced the uptake in the ^211^At-accumulating organs, decreasing the absorbed dose by 55–82%. For the blood and bone marrow, however, a small increase (2–8%) was observed. For tumors, the uptake at 5 hpi was decreased when NaClO_4_ was given. While this decrease was not significant, it contributed to a 13% decrease in tumor dose. There was no gain in the therapeutic window (blood/bone marrow vs tumor) when NaClO_4_ was given and the rationale for pre-treatment could be questioned. The gain will depend on which organ is anticipated to be dose-limiting, but most certainly, bone marrow is one of limiting organs. We have previously estimated the maximum tolerance dose (MTD) for the kidneys following systemic α-RIT with ^211^At in mice [29] and reported a MTD of 10 Gy. In similar, we reported that the total absorbed dose needed for the eradication of macrotumors [21] was estimated to be 10 Gy, also. These numbers can be used as an example to compare the therapeutic window with or without NaClO_4_ pre-treatment in the current study, i.e., anticipating the kidneys as the dose-limiting organ. Without pre-treatment, a total injected activity of 4.5 MBq would be required to reach a tumor dose of 10 Gy, which in turn would give the kidneys a dose of 9.5 Gy. With NaClO_4_ pre-treatment given, the corresponding numbers would be 5.2 MBq injected and 8.9 Gy to the kidneys, i.e., a 7% gain in the therapeutic window. However, at this point, the organ tolerance doses for systemic α-irradiation are largely unknown as are the respective normal organ RBEs (relative biological effectiveness). We have observed (unpublished data) that apart from bone marrow and kidneys, also the lungs could be a dose-liming organ in TAT. Even though the whole-body payload from α-irradiation was significantly decreased by pre-treatment with NaClO_4_ in our study, more studies are needed to evaluate the potential value of blocking agents. It should be noted that the absorbed doses discussed above were not corrected for any RBE of α-radiation, but a factor of 5 is generally accepted.

Among the α-emitters of interest for human use, ^211^At is a main candidate and we have explored it continuously for clinical use in α-RIT of ovarian cancer [30–32]. Astatine-211 offers a theranostic approach to TAT since the decay involves the emission of K X-rays (77–93 keV) allowing for in vivo γ-quantification by planar and SPECT imaging. Another advantage is that ^211^At does not have a series of α-particle emitting daughters in its decay chain. This can be of importance for accurate risk analysis and dose-planning, especially for TAT regimens with curative intent.

Their rationale for directly cell-targeted TAT of mCRPC is strong as indicated by the promising results with anti-PSMA ligands labeled with α-emitters [2–9]. While PSMA is detected on the majority of prostate cancer cases, the expression can be heterogenic at all levels; patient, lesion [33] and circulating tumor cells (CTCs). One study reported that the fraction of patients having a PSMA-positive phenotype in PC relapses was 82.8% [34], i.e., 17% would be PSMA-negative. A similar fraction (16%) was found not eligible for ^177^Lu-PSMA-617 therapy [10], due to low PSMA-expression or FDG discordance. An imaging study with ^89^Zr-J591 reported that 36% of pathologic positive lesion sites were PSMA-negative [11]. Another study compared imaging with biomarker analysis of CTCs showing that while nearly 100% of the lesions (bone and soft tissue ) were PSMA-positive, only 43% of the patients had PSMA-positive CTCs [35]. Further, it has been reported that for mCRPC patients who progress after conventional treatments, a low PSMA-expression (or discordant PET/CT) correlated with poor prognosis and short surviva l[36]. In a study using immunohistochemistry to analyze the expression of both PSMA and PSCA on patient lymph nodes and bone metastases, both antigens had high overall staining frequency (94–100%), and in some samples, PSCA had a higher score than PSMA [37]. All this underlines the need for other targets that can be complementary to, or even combined with, PSMA-targeting. In fact, dual targeting of two antigens, e.g., PSMA and PSCA, either as a cocktail or using a bispecific targeting agent, could potentiate future treatments of mCRPC further, as indicated by [38].

## Conclusion

We evaluated the concept of systemic TAT for treatment of mCRPC. Using a fractionated regimen of α-RIT with ^211^At-labeled anti-PSCA A11 minibody, we found strong growth inhibition on both macrotumors and intratibial microtumors. These findings are conceptually promising for systemic TAT of mCRPC and warrant further investigations of ^211^At-labeled vectors, including anti-PSCA antibodies and other molecules having PSCA as the target for therapy. Such investigations should include further optimization of the therapeutic window, e.g., by implementing pre-targeting (PRIT) or by altering the size of the targeting vector.

## Supplementary information

**Additional file 1: Figure S1.** Aggregate and fragmentation analysis was performed before and after radiolabeling, using size exclusion liquid chromatography, FPLC (Superdex 200).

**Additional file 2: Figure S2.** The immunoreactive fraction (IRF) of the minibody after radiolabeling with ^211^At was analyzed in a viable cell assay previously described. Serial 1:2 dilutions of PC3- PSCA cell suspensions (0.15625 to 10 million cells per mL) were incubated with 5 ng ^211^At- A11.1 at 8ºC. After a 3 hour incubation, centrifugation and washing of the pellets, the IRF was calculated from the double-inverse plot of specific binding (B/T) over cell concentration. The plot above corresponds to a IRF of 0.67.

**Additional file 3: Figure S3.** Blood counts as a function of days after i.v. treatment with 211At-A11 minibody at different injected activities. (a) white blood cell, (b) platelets, (c) red blood cells and (d) hemoglobin. Data points represent the mean of 5 mice.

**Additional file 4: Figure S4.** Data used for screening of the PC3-PSCA cell clones. (a) mRNA-quantifications of the PSCA-expression of 11 different PC3-transfected cell-clones. (b) Cell binding assay data used for screening of 4 of the PSCA-PC3-clones with the highest PSCA-expression.

**Additional file 5: Figure S5.** Comparative biodistribution of minibody A11 labeled with ^211^At (m-Me-ATE) versus ^125^I (Iodogen) for blood concentration (a) and uptake in s.c.-PC3-PSCA-macrotumors.

**Additional file 6: Figure S6.** (a) Comparison of bone marrow uptake at 1 hpi of ^211^At-labeled minibody A11 labeled with the m-Me-ATE-method (described in the paper) as compared to labeling with the B10 boron cage method [28]. (b) Bone marrow-to-Blood-ratio (BMBLR) at 1 hpi. Labeling procedure B-10 .Briefly, the B-10 derivative was conjugated to the antibody as follows: a 10 time excess of the B-10 derivative was added to the antibody at a concentration of 3-4 mg/ml in carbonate buffer pH 8.5. The reaction was allowed to proceed over night at gentle agitation. The conjugated antibody was isolated by passage over a NAP-5 column. The column was eluted with PBS. A dry residue of 211At was activated by 10 μl, 2 nmole NIS in methanol/1% acetic acid. To the At-211/ NIS was then 100 μg, 200 μl B-10-Antibody added under agitation. After 1 minute the reaction was stopped by adding 0.8 μmole sodium ascorbate. Finally, the labeled antibody was isolated by size exclusion chromatography on NAP-5 column. Radiochemical yields was in the range of 65-80% .

## Data Availability

The data sets used in the current study are available from the corresponding author on reasonable request.

## Abbreviations

BM: Bone marrow
BMBLR: Bone marrow-to-blood-ratio
HGB: Hemoglobin
hpi: Hours post-injection
i.t.: Intratibial
i.v.: Intravenously
IRF: Immunoreactive fraction
mCRPC: Metastatic castration-resistant prostate cancer
MTA: Maximum tolerable activity
PC: Prostate cancer
PLT: Platelets
PRIT: Pre-targeted radioimmunotherapy
PSCA: Prostate stem cell antigen
PSMA: Prostate-specific membrane antigen
RBC: Red blood cells
s.c.: Subcutaneous
TAT: Targeted alpha therapy
TFF: Tumor-free fraction
WBC: White blood cells
α-RIT: α-radioimmunotherapy

## References

[1] Sartor Oliver, Sharma Deepali (2018). Radium and other alpha emitters in prostate cancer. Translational Andrology and Urology.

[2] Kratochwil C, Bruchertseifer F, Giesel FL, Weis M, Verburg FA, Mottaghy F (2016). 225Ac-PSMA-617 for PSMA-Targeted alpha-radiation therapy of metastatic castration-resistant prostate cancer. Journal of nuclear medicine : official publication, Society of Nuclear Medicine..

[3] Kratochwil C, Bruchertseifer F, Rathke H, Bronzel M, Apostolidis C, Weichert W (2017). Targeted alpha-therapy of metastatic castration-resistant prostate cancer with (225)Ac-PSMA-617: Dosimetry Estimate and Empiric Dose Finding. Journal of nuclear medicine : official publication, Society of Nuclear Medicine..

[4] Kratochwil C, Bruchertseifer F, Rathke H, Hohenfellner M, Giesel FL, Haberkorn U (2018). Targeted alpha-therapy of metastatic castration-resistant prostate cancer with (225)Ac-PSMA-617: Swimmer-plot analysis suggests efficacy regarding duration of tumor Control. Journal of nuclear medicine : official publication, Society of Nuclear Medicine..

[5] Rathke H, Kratochwil C, Hohenberger R, Giesel FL, Bruchertseifer F, Flechsig P (2019). Initial clinical experience performing sialendoscopy for salivary gland protection in patients undergoing (225)Ac-PSMA-617 RLT. European journal of nuclear medicine and molecular imaging..

[6] Sathekge M, Bruchertseifer F, Knoesen O, Reyneke F, Lawal I, Lengana T (2019). (225)Ac-PSMA-617 in chemotherapy-naive patients with advanced prostate cancer: a pilot study. European journal of nuclear medicine and molecular imaging..

[7] Kratochwil C, Schmidt K, Afshar-Oromieh A, Bruchertseifer F, Rathke H, Morgenstern A (2018). Targeted alpha therapy of mCRPC: Dosimetry estimate of (213)Bismuth-PSMA-617. European journal of nuclear medicine and molecular imaging..

[8] Kelly James M., Amor-Coarasa Alejandro, Ponnala Shashikanth, Nikolopoulou Anastasia, Williams Clarence, Thiele Nikki A., Schlyer David, Wilson Justin J., DiMagno Stephen G., Babich John W. (2018). A Single Dose of 225Ac-RPS-074 Induces a Complete Tumor Response in an LNCaP Xenograft Model. Journal of Nuclear Medicine.

[9] Kiess AP, Minn I, Vaidyanathan G, Hobbs RF, Josefsson A, Shen C (2016). (2S)-2-(3-(1-Carboxy-5-(4-211At-Astatobenzamido)Pentyl)Ureido)-Pentanedioic Acid for PSMA-Targeted alpha-Particle Radiopharmaceutical Therapy. Journal of nuclear medicine : official publication, Society of Nuclear Medicine..

[10] Miyahira AK, Pienta KJ, Morris MJ, Bander NH, Baum RP, Fendler WP (2018). Meeting report from the Prostate Cancer Foundation PSMA-directed radionuclide scientific working group. The Prostate..

[11] Pandit-Taskar N, O'Donoghue JA, Durack JC, Lyashchenko SK, Cheal SM, Beylergil V (2015). A phase I/II study for analytic validation of 89Zr-J591 ImmunoPET as a Molecular Imaging Agent for Metastatic Prostate Cancer. Clin Cancer Res..

[12] Gu Z, Thomas G, Yamashiro J, Shintaku IP, Dorey F, Raitano A (2000). Prostate stem cell antigen (PSCA) expression increases with high gleason score, advanced stage and bone metastasis in prostate cancer. Oncogene..

[13] Olafsen T, Gu Z, Sherman MA, Leyton JV, Witkosky ME, Shively JE (2007). Targeting, imaging, and therapy using a humanized antiprostate stem cell antigen (PSCA) antibody. Journal of immunotherapy..

[14] Lepin EJ, Leyton JV, Zhou Y, Olafsen T, Salazar FB, McCabe KE (2010). An affinity matured minibody for PET imaging of prostate stem cell antigen (PSCA)-expressing tumors. European journal of nuclear medicine and molecular imaging..

[15] Knowles SM, Zettlitz KA, Tavare R, Rochefort MM, Salazar FB, Stout DB (2014). Quantitative immunoPET of prostate cancer xenografts with 89Zr- and 124I-labeled anti-PSCA A11 minibody. Journal of nuclear medicine : official publication, Society of Nuclear Medicine..

[16] Leyton JV, Olafsen T, Lepin EJ, Hahm S, Bauer KB, Reiter RE (2008). Humanized radioiodinated minibody for imaging of prostate stem cell antigen-expressing tumors. Clin Cancer Res..

[17] Reiter RE, Gu Z, Watabe T, Thomas G, Szigeti K, Davis E (1998). Prostate stem cell antigen: a cell surface marker overexpressed in prostate cancer. Proceedings of the National Academy of Sciences of the United States of America..

[18] Lindegren S, Back T, Jensen HJ (2001). Dry-distillation of astatine-211 from irradiated bismuth targets: a time-saving procedure with high recovery yields. Applied radiation and isotopes : including data, instrumentation and methods for use in agriculture, industry and medicine..

[19] Lindegren S, Frost S, Back T, Haglund E, Elgqvist J, Jensen H (2008). Direct procedure for the production of 211At-labeled antibodies with an epsilon-lysyl-3-(trimethylstannyl)benzamide immunoconjugate. Journal of nuclear medicine : official publication, Society of Nuclear Medicine..

[20] Hagberg Thulin Malin, Jennbacken Karin, Damber Jan-Erik, Welén Karin (2013). Osteoblasts stimulate the osteogenic and metastatic progression of castration-resistant prostate cancer in a novel model for in vitro and in vivo studies. Clinical & Experimental Metastasis.

[21] Back T, Chouin N, Lindegren S, Kahu H, Jensen H, Albertsson P (2017). Cure of Human Ovarian Carcinoma Solid Xenografts by Fractionated alpha-Radioimmunotherapy with 211At-MX35-F(ab')2: Influence of Absorbed Tumor Dose and Effect on Long-Term Survival. Journal of nuclear medicine : official publication, Society of Nuclear Medicine..

[22] Bäck T, Jacobsson L (2010). The Alpha-Camera: A quantitative digital autoradiography technique using a charge-coupled device for ex vivo high-resolution bioimaging of alpha-particles. Journal of nuclear medicine : official publication, Society of Nuclear Medicine..

[23] Ferlay J, Steliarova-Foucher E, Lortet-Tieulent J, Rosso S, Coebergh JW, Comber H (2013). Cancer incidence and mortality patterns in Europe: estimates for 40 countries in 2012. European journal of cancer..

[24] Sartor O, de Bono JS (2018). Metastatic Prostate Cancer. The New England journal of medicine..

[25] Fukumura D, Jain RK (2007). Tumor microvasculature and microenvironment: targets for anti-angiogenesis and normalization. Microvasc Res..

[26] Chouin N, Lindegren S, Jensen H, Albertsson P, Back T (2012). Quantification of activity by alpha-camera imaging and small-scale dosimetry within ovarian carcinoma micrometastases treated with targeted alpha therapy. The quarterly journal of nuclear medicine and molecular imaging : official publication of the Italian Association of Nuclear Medicine..

[27] Chouin N, Lindegren S, Frost SH, Jensen H, Albertsson P, Hultborn R (2013). Ex vivo activity quantification in micrometastases at the cellular scale using the alpha-camera technique. Journal of nuclear medicine : official publication, Society of Nuclear Medicine..

[28] Orozco JJ, Back T, Kenoyer A, Balkin ER, Hamlin DK, Wilbur DS (2013). Anti-CD45 radioimmunotherapy using (211)At with bone marrow transplantation prolongs survival in a disseminated murine leukemia model. Blood..

[29] Bäck T, Haraldsson B, Hultborn R, Jensen H, Johansson ME, Lindegren S (2009). Glomerular filtration rate after alpha-radioimmunotherapy with 211At-MX35-F(ab')2: a long-term study of renal function in nude mice. Cancer Biother Radiopharm..

[30] Andersson H, Cederkrantz E, Back T, Divgi C, Elgqvist J, Himmelman J (2009). Intraperitoneal alpha-particle radioimmunotherapy of ovarian cancer patients: pharmacokinetics and dosimetry of (211)At-MX35 F(ab')2--a phase I study. Journal of nuclear medicine : official publication, Society of Nuclear Medicine..

[31] Cederkrantz E, Andersson H, Bernhardt P, Back T, Hultborn R, Jacobsson L (2015). Absorbed Doses and Risk Estimates of (211)At-MX35 F(ab')2 in Intraperitoneal Therapy of Ovarian Cancer Patients. International journal of radiation oncology, biology, physics..

[32] Hallqvist Andreas, Bergmark Karin, Bäck Tom, Andersson Håkan, Dahm-Kähler Pernilla, Johansson Mia, Lindegren Sture, Jensen Holger, Jacobsson Lars, Hultborn Ragnar, Palm Stig, Albertsson Per (2019). Intraperitoneal α-Emitting Radioimmunotherapy with 211At in Relapsed Ovarian Cancer: Long-Term Follow-up with Individual Absorbed Dose Estimations. Journal of Nuclear Medicine.

[33] Mannweiler S, Amersdorfer P, Trajanoski S, Terrett JA, King D, Mehes G (2009). Heterogeneity of prostate-specific membrane antigen (PSMA) expression in prostate carcinoma with distant metastasis. Pathol Oncol Res..

[34] Afshar-Oromieh A, Avtzi E, Giesel FL, Holland-Letz T, Linhart HG, Eder M (2015). The diagnostic value of PET/CT imaging with the (68)Ga-labelled PSMA ligand HBED-CC in the diagnosis of recurrent prostate cancer. European journal of nuclear medicine and molecular imaging..

[35] Morris Michael J., Vogelzang Nicholas J., Sartor A. Oliver, Armour Alison A., Messmann Richard Adam, Groaning Michael, Robarts Adam, Tolcher Anthony W., Gordon Michael S., Babiker Hani M., Kuo Phillip, Kearney Megan, Jendrisak Adam, Wang Yipeng, Landers Mark Andrew, Petrylak Daniel Peter (2018). PSMA heterogeneity analysis in patients with metastatic castrate-resistant prostate cancer (mCRPC): Imaging versus CTCs. Journal of Clinical Oncology.

[36] Thang Sue Ping, Violet John A, Sandhu Shahneen Kaur, Iravani Amir, Akhurst Timothy J., Ravi Kumar Aravind, Kong Grace, Williams Scott, Hicks Rodney J, Hofman Michael (2018). The poor outcome of patients with mCRPC whom were deemed ineligible for PSMA theranostics based on molecular imaging characteristics. Journal of Clinical Oncology.

[37] Ananias HJ, van den Heuvel MC, Helfrich W, de Jong IJ (2009). Expression of the gastrin-releasing peptide receptor, the prostate stem cell antigen and the prostate-specific membrane antigen in lymph node and bone metastases of prostate cancer. The Prostate..

[38] Arndt C, Feldmann A, Koristka S, Cartellieri M, Dimmel M, Ehninger A (2014). Simultaneous targeting of prostate stem cell antigen and prostate-specific membrane antigen improves the killing of prostate cancer cells using a novel modular T cell-retargeting system. The Prostate..

