# Supplementary figures and images for: Targeted alpha therapy with astatine-211-labeled anti-PSCA A11 minibody shows antitumor efficacy in prostate cancer xenografts and bone microtumors
Source: EJNMMI Res. 2020 Feb 11;10:10. doi: 10.1186/s13550-020-0600-z (PMC7013029; doi:10.1186/s13550-020-0600-z)

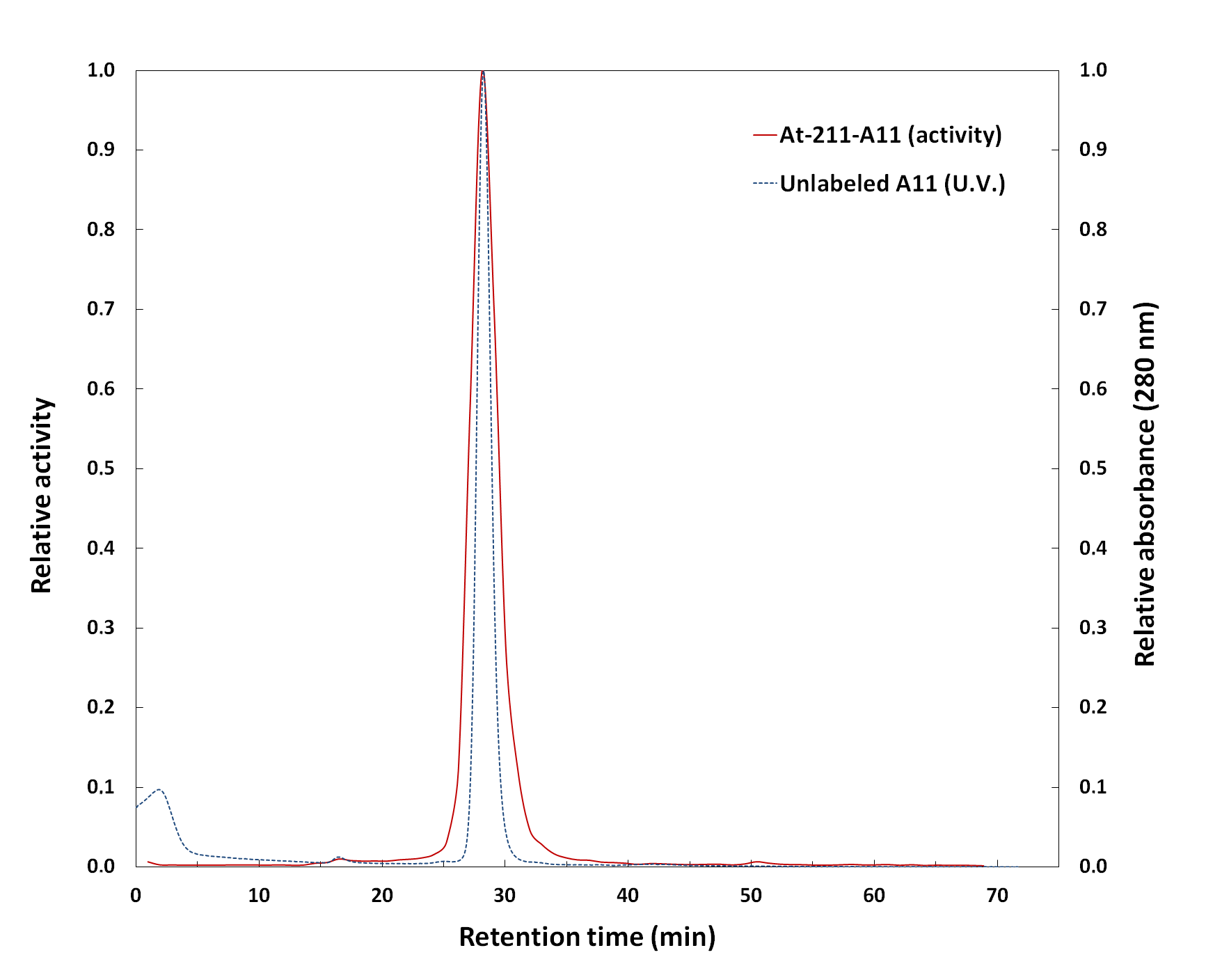

Supplement: Supplementary file 1 — Additional file 1: Figure S1. Aggregate and fragmentation analysis was performed before and after radiolabeling, using size exclusion liquid chromatography, FPLC (Superdex 200). [file 13550_2020_600_MOESM1_ESM.tif]

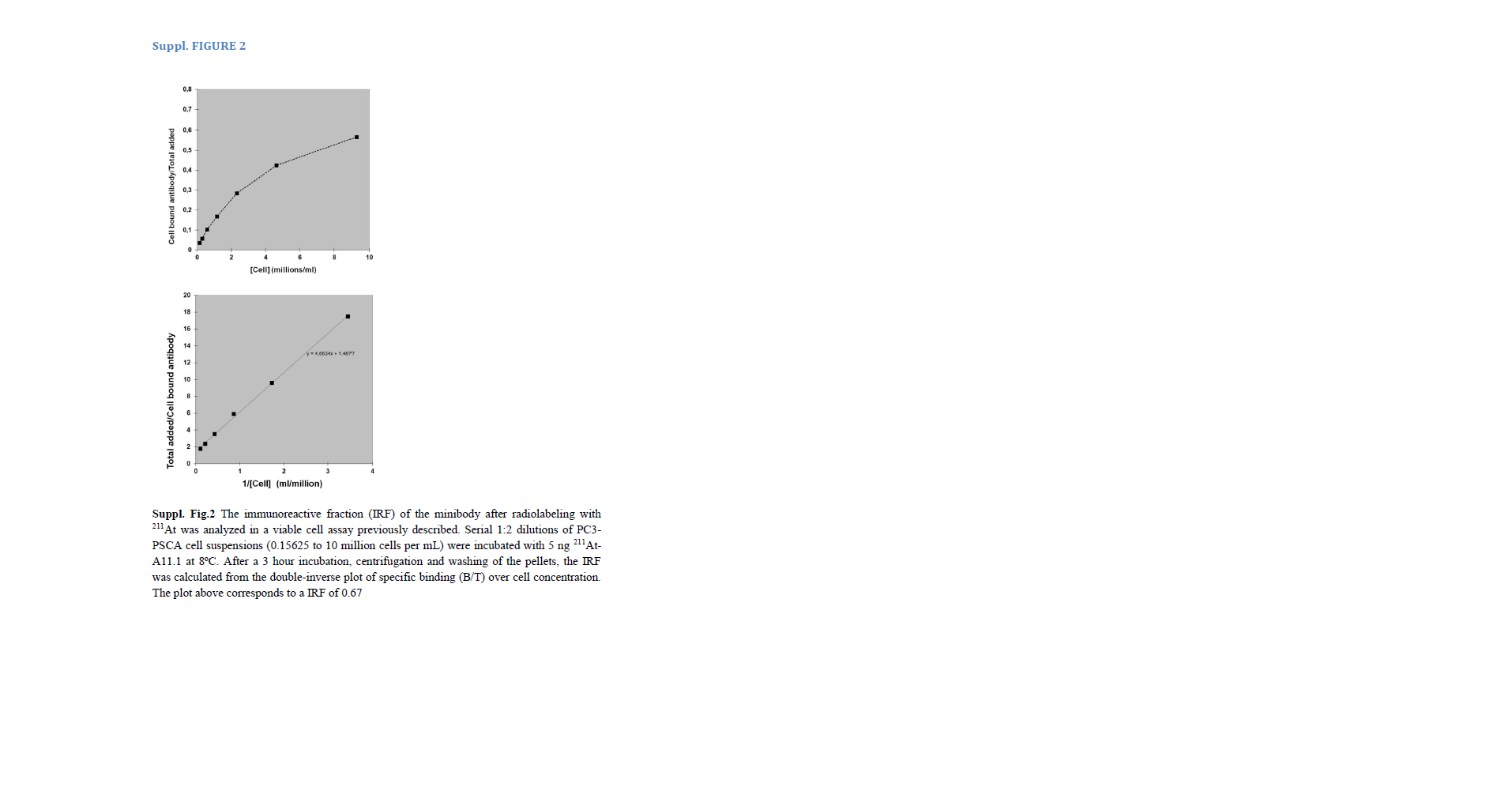

Supplement: Supplementary file 2 — Additional file 2: Figure S2. The immunoreactive fraction (IRF) of the minibody after radiolabeling with 211At was analyzed in a viable cell assay previously described. Serial 1:2 dilutions of PC3- PSCA cell suspensions (0.15625 to 10 million cells per mL) were incubated with 5 ng 211At- A11.1 at 8ºC. After a 3 hour incubation, centrifugation and washing of the pellets, the IRF was calculated from the double-inverse plot of specific binding (B/T) over cell concentration. The plot above corresponds to a IRF of 0.67. [file 13550_2020_600_MOESM2_ESM.tif]

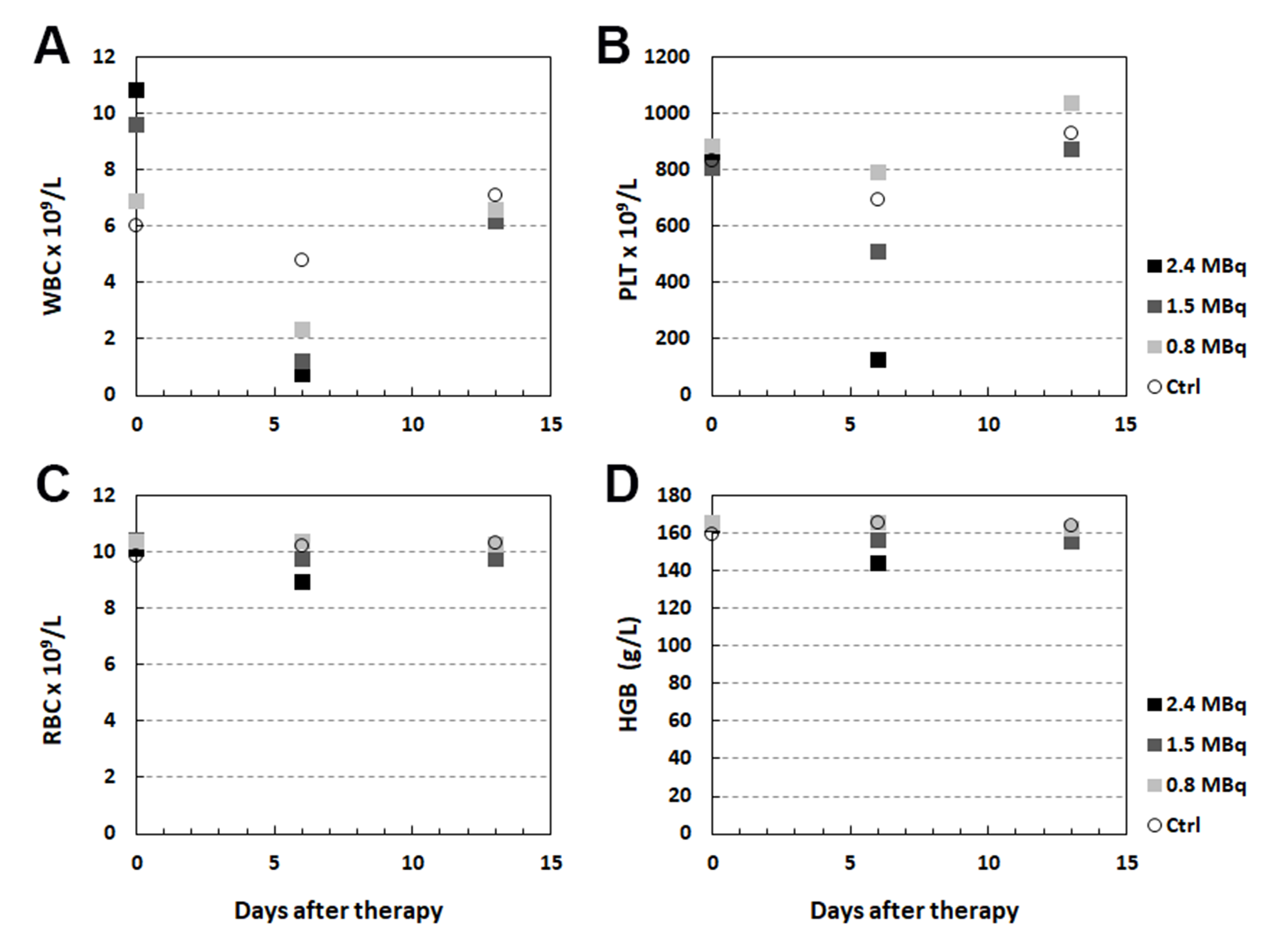

Supplement: Supplementary file 3 — Additional file 3: Figure S3. Blood counts as a function of days after i.v. treatment with 211At-A11 minibody at different injected activities. (a) white blood cell, (b) platelets, (c) red blood cells and (d) hemoglobin. Data points represent the mean of 5 mice. [file 13550_2020_600_MOESM3_ESM.tif]

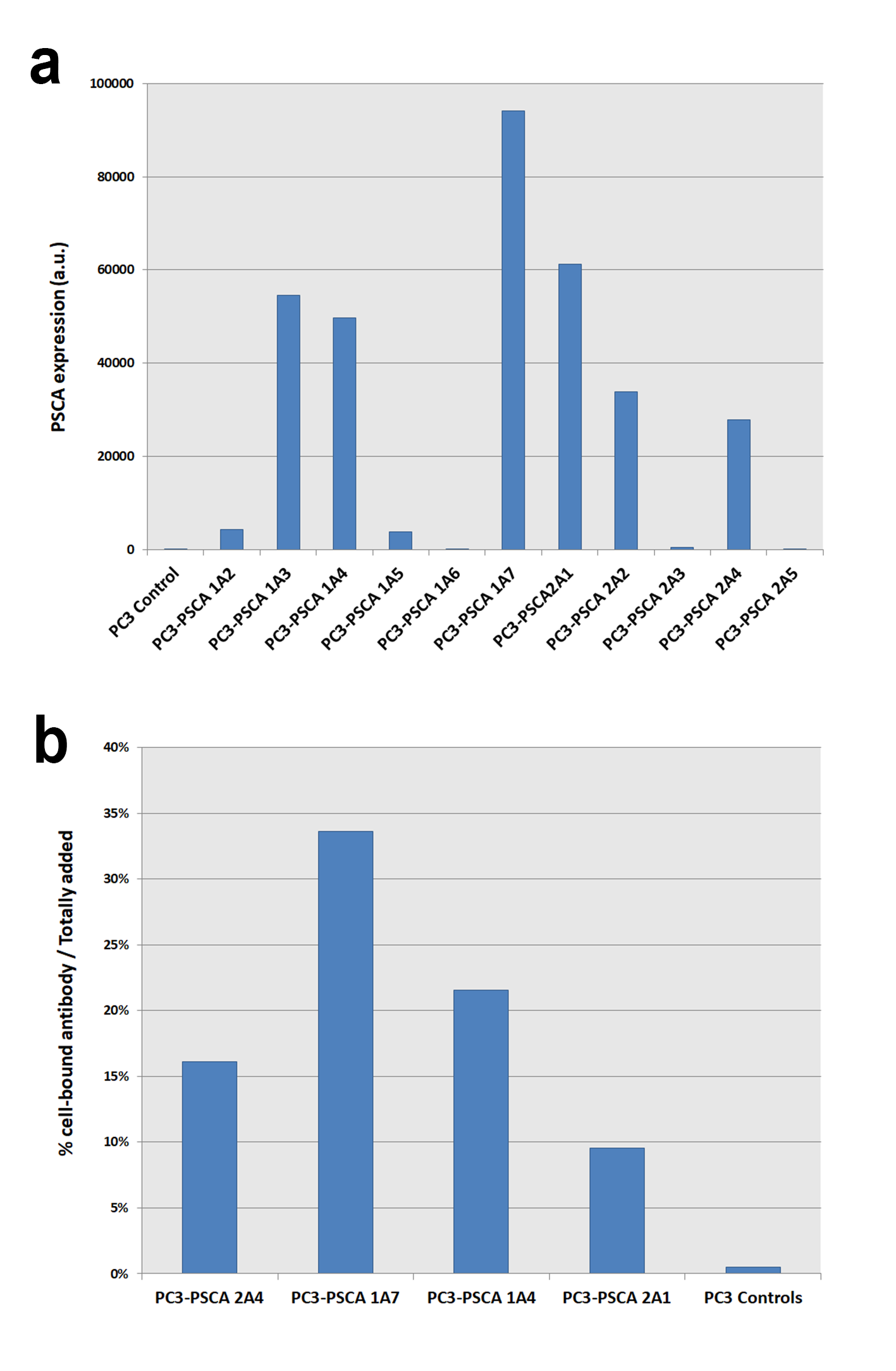

Supplement: Supplementary file 4 — Additional file 4: Figure S4. Data used for screening of the PC3-PSCA cell clones. (a) mRNA-quantifications of the PSCA-expression of 11 different PC3-transfected cell-clones. (b) Cell binding assay data used for screening of 4 of the PSCA-PC3-clones with the highest PSCA-expression. [file 13550_2020_600_MOESM4_ESM.tif]

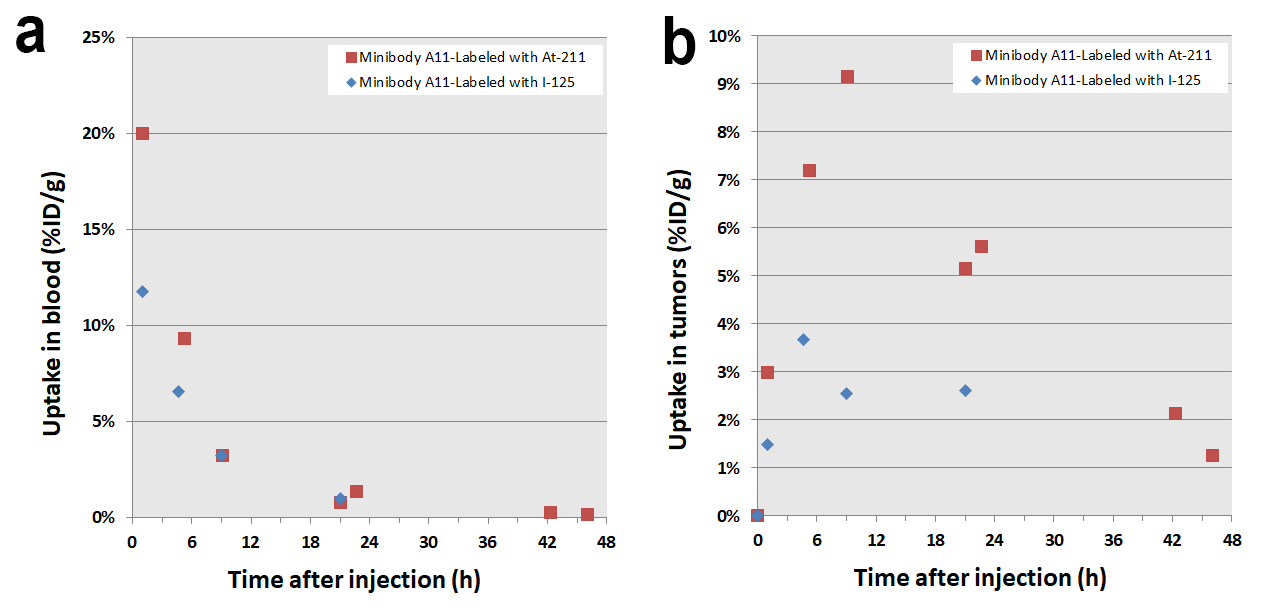

Supplement: Supplementary file 5 — Additional file 5: Figure S5. Comparative biodistribution of minibody A11 labeled with 211At (m-Me-ATE) versus 125I (Iodogen) for blood concentration (a) and uptake in s.c.-PC3-PSCA-macrotumors. [file 13550_2020_600_MOESM5_ESM.tif]

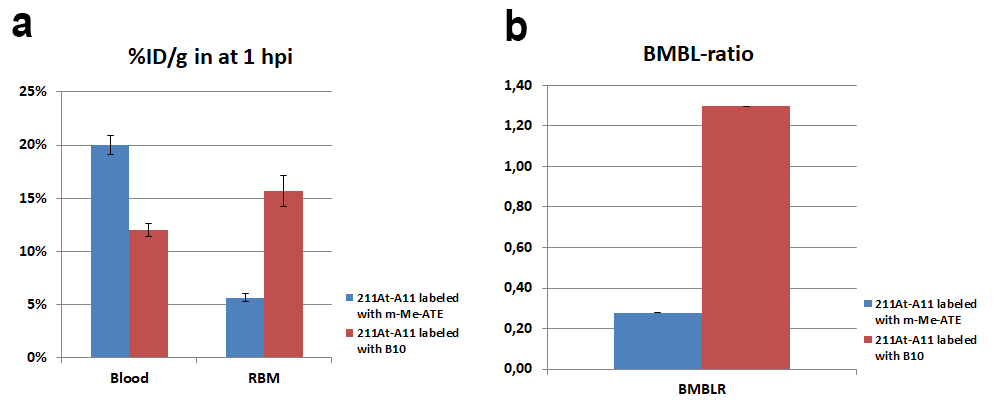

Supplement: Supplementary file 6 — Additional file 6: Figure S6. (a) Comparison of bone marrow uptake at 1 hpi of 211At-labeled minibody A11 labeled with the m-Me-ATE-method (described in the paper) as compared to labeling with the B10 boron cage method [28]. (b) Bone marrow-to-Blood-ratio (BMBLR) at 1 hpi. Labeling procedure B-10 .Briefly, the B-10 derivative was conjugated to the antibody as follows: a 10 time excess of the B-10 derivative was added to the antibody at a concentration of 3-4 mg/ml in carbonate buffer pH 8.5. The reaction was allowed to proceed over night at gentle agitation. The conjugated antibody was isolated by passage over a NAP-5 column. The column was eluted with PBS. A dry residue of 211At was activated by 10 μl, 2 nmole NIS in methanol/1% acetic acid. To the At-211/ NIS was then 100 μg, 200 μl B-10-Antibody added under agitation. After 1 minute the reaction was stopped by adding 0.8 μmole sodium ascorbate. Finally, the labeled antibody was isolated by size exclusion chromatography on NAP-5 column. Radiochemical yields was in the range of 65-80% . [file 13550_2020_600_MOESM6_ESM.tif]
